# Rodent Ulcer and Epithelioma

**Published:** 1881-12

**Authors:** 


### RODENT ULCER AND EPITHELIOMA.

Rodent ulcer and epithelioma are undoubtedly closely allied affections, although rodent ulcer differs in some important respects from epithelioma.

Rodent ulcer does not affect lymphatic glands, whilst epithelioma does, and often at an early stage of the disease.

Rodent ulcer is a rather dry ulceration with only little secretion and no fetor, and the granulations are small. In epithelioma the secretion from the ulcerated surface is abundant and fœted, and the granulations large, exuberant, and often in bosses.

Rodent ulcer is confined to the upper part of the face, whilst epithelioma, although it has a preference for certain localities, may under certain conditions attack any part of the body.

The points of resemblance between rodent ulcer and epithelioma are:

Both rodent ulcer and epithelioma are new growths, composed mainly of epithelial cells; and the new growth is only part involved in the ulceration. As the growth increases and includes more of the skin and deeper tissues, so the ulceration continues to extend; but the ulceration does not go beyond the new deposit.

On one point I am quite settled, that rodent ulcer, if left, will in time so change its character as to become true epithelioma. I cannot say whether this change is due to a mere progress of the disease, or whether it is that rodent ulcer is peculiarly apt to have superadded to it the characters of epithelioma after the same manner as that of old standing ulcers, unhealed wounds, or scars, or other simple sores that become epitheliomatous—Lawson, *Ophthalmic Hospital Reports*, Vol. x., part ii., 1881.

